# Viral pathogens in the etiology of acute respiratory infections in Bulgaria during the 2024–2025 season and genetic diversity of circulating influenza viruses

**DOI:** 10.3389/fmicb.2026.1785399

**Published:** 2026-04-16

**Authors:** Neli Korsun, Ivelina Trifonova, Diana Pavlova, Iliana Grigorova, Yordanka Uzunova, Ivan Ivanov, Daniel Ivanov, Petar Velikov, Silvia Voleva, Iva Christova

**Affiliations:** 1 National Laboratory “Influenza and ARD”, Department of Virology, National Center of Infectious and Parasitic Diseases, Sofia, Bulgaria; 2 Center of Competence “ImmunoPathogen”, Sofia, Bulgaria; 3 University Hospital “Lozenetz”, Sofia, Bulgaria; 4 Department for Infectious Diseases, Parasitology and Tropical Medicine, Medical University of Sofia, Sofia, Bulgaria; 5 Infectious Diseases Hospital “Prof. Ivan Kirov”, Sofia, Bulgaria

**Keywords:** acute respiratory infection, amino acid substitution, co-infection, genetic characterization, influenza virus, vaccine, whole-genome sequencing

## Abstract

**Background:**

Respiratory viruses are major contributors to global morbidity and mortality, with influenza viruses having a significant clinical and epidemiological impact. In this study, we examined the virological and epidemiological aspects of influenza infection in comparison with those of other respiratory infections. We further assessed the genetic diversity of circulating influenza viruses and their susceptibility to antivirals.

**Methods:**

A total of 2,981 nasopharyngeal specimens from patients with acute respiratory illness were tested for 13 different respiratory viruses using multiplex real-time PCR. Representative influenza strains underwent whole-genome sequencing, phylogenetic and amino acid analyses.

**Results:**

At least one respiratory virus was detected in 1,635 (54.8%) patients, and 182 (6.1%) had co-infections involving two to four viruses, including six cases of SARS-CoV-2 and influenza co-infection. Influenza A(H3N2) and B/Victoria lineage viruses predominated, with positivity rates of 12.1 and 10.9%, respectively, followed by rhinoviruses (9.0%), whereas SARS-CoV-2 circulation remained low (3.7%). Influenza infections were most prevalent among children aged 5–14 years. Phylogenetic analysis identified multiple genetic subclades, including C.1.9, C.1.9.1, C.1.9.3, C.1.9.4 and D.5 for A(H1N1)pdm09; J.2, J.2.1, and J.2.2 for A(H3N2), and C.5.1, C.5.6, C.5.6.1, and C.5.7 for B/Victoria lineage. Numerous amino acid substitutions were observed across viral proteins, including within hemagglutinin antigenic sites compared to vaccine strains.

**Conclusion:**

The 2024–2025 influenza season in Bulgaria was characterized by high influenza activity and substantial genetic heterogeneity of circulating strains. These findings underline the importance of continuous molecular surveillance to monitor viral evolution, inform vaccine strain selection, and guide national public health strategies.

## 1 Introduction

Viral acute respiratory infections (ARIs) are the leading causes of morbidity and mortality worldwide. They represent a major disease burden, especially in young children, the elderly, and people with chronic diseases (Uyeki et al., 2022). The high transmission of respiratory viruses and their remarkable adaptability to host organisms, coupled with rapid replication and evolution, facilitate their widespread circulation. Additionally, the short incubation periods of these infections, combined with human behavior, considerably contribute to their spread.

Multiple viruses cause ARIs, including influenza viruses, rhinoviruses (RVs), coronaviruses (CoVs), respiratory syncytial virus (RSV), human metapneumovirus (hMPV), parainfluenza viruses (PIVs), adenoviruses (AdVs), and human bocavirus (BoV) (Contes and Liu, 2025). Among these, influenza viruses have a particularly substantial impact on public health due to their potential to cause severe complications and their ability to cause annual epidemics and sporadic pandemics (Cassini et al., 2018). Seasonal influenza epidemics are associated with approximately 1 billion clinical cases worldwide, including 3–5 million cases of severe illness, and 290,000–650,000 deaths annually (WHO, 2024a). In Bulgaria, influenza epidemics typically occur in January–February and represent a great burden on the healthcare system.

Since the 2009–2010 influenza pandemic, two subtypes of influenza A viruses, A(H1N1)pdm09 and A(H3N2), and two genetic lineages of B viruses, B/Victoria and B/Yamagata, have circulated globally (Uyeki et al., 2022). However, no confirmed cases of B/Yamagata lineage infection have been reported worldwide since March 2020 (Koutsakos et al., 2021).

Influenza viruses A and B belong to the family *Orthomyxoviridae* and have a genome consisting of eight segments of single-stranded RNA with negative polarity. Each segment encodes at least one protein: PB2, PB1, PA, HA, NP, NA, M1/M2, and NS1/NS2. The surface glycoproteins hemagglutinin (HA) and neuraminidase (NA) play crucial roles in the initial and final stages of viral replication, respectively. HA recognizes cell sialic acid receptors and facilitates the entry of the virus into host cells, whereas NA aids in the release of new virus particles from infected cells. HA and NA rapidly mutate under the pressure of the host’s immune system, resulting in the emergence of new genetic variants. HA is a key antigen of the virus and serves as the primary target for host-neutralizing antibodies. Mutations in the HA1 region of this protein, particularly in the binding sites of neutralizing antibodies (known as antigenic sites), can alter the antigenic properties of the virus. This process, referred to as antigenic drift, is an important mechanism by which the virus evades neutralizing antibodies generated from prior infections or vaccinations (Wei et al., 2020).

Although vaccination remains the primary measure for controlling influenza infection, antiviral drugs are important therapeutic options. In particular, two classes of antiviral drugs (NA inhibitors and the PA inhibitor baloxavir) exhibit high activity against recently circulating viruses. While resistance-associated mutations have been identified in NA and PA genes, the prevalence of influenza viruses with reduced susceptibility to recommended antivirals remains low (Hussain et al., 2025).

The National Reference Laboratory (NRL) “Influenza and ARD” in Sofia conducts year-round surveillance of influenza and other respiratory viruses in accordance with WHO recommendations (Korsun et al., 2023). Due to the high genetic variability of influenza viruses and their continuous antigenic evolution, data on circulating strains are season-specific and cannot be extrapolated from previous years. Therefore, systematic monitoring of virological, epidemiological, and genetic characteristics is essential for assessing disease burden, informing vaccine strain selection, and guiding national public health strategies.

The present study aimed to investigate the virological and epidemiological characteristics of influenza viruses circulating in Bulgaria during the 2024–2025 season in comparison with other respiratory viruses. Additionally, we sought to explore influenza virus involvement in co-infections, assess their susceptibility to antivirals, genetic diversity, and amino-acid variations across viral proteins.

## 2 Materials and methods

This prospective observational study was conducted within the framework of the Bulgarian national influenza surveillance program during the 2024–2025 influenza season. Patients presenting with symptoms of ARI in primary healthcare facilities and hospitals across the country were eligible for inclusion. Between October 2024 and May 2025, respiratory samples were collected as part of the routine national influenza surveillance activities. Anonymized data were prospectively analyzed for research purposes. The study followed the European Centre for Disease Prevention and Control (ECDC) definition of ARI, which includes: (1) an acute onset of symptoms; (2) the presence of at least one of four respiratory symptoms (cough, sore throat, nasal congestion, or wheezing); and (3) a clinical assessment by a physician that indicates a suspected infectious illness (ECDC, 2025a). Both outpatients and hospitalized patients were enrolled.

Nasopharyngeal and oropharyngeal swabs were obtained from outpatients during medical visits or from inpatients within the first 24 h of hospital admission. These swabs were placed in 2 mL of viral transport medium and transported to the NRL “Influenza and ARD” in insulated cool boxes with ice packs. If immediate delivery was not possible, specimens were stored in healthcare facilities at 2–8 °C for up to 24 h. The specimens were processed and tested on the same day they arrived at the laboratory, and aliquots of the primary samples were preserved at −80 °C to maintain their integrity.

### 2.1 Molecular detection of respiratory viruses

Viral nucleic acids were extracted from 400 μL of speciment using an ExiPrep Dx Viral DNA/RNA kit on an ExiPrep 16DX instrument (Bioneer, Republic of Korea) with an elution volume of 100 μL. The FluSC2 Multiplex Real-Time RT-PCR Kit provided by the International Reagent Resource (IRR) (USA) was used in combination with the Applied Biosystems TaqPath™ 1-Step Multiplex Master Mix to detect influenza A/B viruses and SARS-CoV-2 simultaneously. Human RNase P was included as an internal control (Shu et al., 2021). Samples negative for the RNase P assay were excluded.

Three multiplex real-time PCR assays were performed using primers and probes as previously described to detect the eight common non-influenza respiratory viruses: RSV, hMPV, PIV 1/2/3, RV, AdV, and BoV (Kodani et al., 2011). Reactions included virus-specific primers and TaqMan probes labeled with different fluorescent dyes, along with appropriate positive and negative controls. Amplifications were performed on a CFX96 thermal cycler (Bio-Rad Laboratories, Inc., Singapore) under the following conditions: 2 min at 25 °C, followed by 15 min at 50 °C, 2 min at 95 °C, and then 45 cycles of 15 s at 95 °C and 30 s at 55 °C. Specimens were considered positive if the cycle threshold (Ct) value was ≤38.

Influenza-positive samples were subtyped by real-time RT-PCR with the SuperScript III Platinum One-Step qRT-PCR kit (Invitrogen, Thermo Fisher Scientific, Waltham, MA, USA), and pathogen-specific primer/probe sets were provided by IRR. The amplification procedure followed the protocol recommended by the Centers for Disease Control and Prevention (CDC) (Atlanta, GA, USA) (Shu et al., 2011).

### 2.2 Influenza virus isolation

Influenza virus-positive samples identified by PCR were isolated and cultured in Madin-Darby canine kidney (MDCK) cells (for influenza A/H1N1/pdm09 and influenza type B), and MDCK-SIAT cells (for influenza A/H3N2) as previously described (WHO, 2011). Viral isolates were stored at −70 °C.

### 2.3 Determination of antiviral susceptibility

Phenotypic susceptibility of influenza viruses to the neuraminidase (NA) inhibitor oseltamivir was determined using the NA-Fluor Influenza Neuraminidase Assay Kit (Applied Biosystems, USA) and the Synergy HTX^®^ Multimode Reader. The NA enzymatic activity of each virus was measured using the fluorescent substrate 2′-(4-methylumbelliferyl)-*α*-D-N-acetylneuraminic acid (MUNANA) to determine the optimal virus sample dilution. Addition of an inhibitor enabled the determination of the drug concentration required to inhibit the enzyme activity by 50% (IC50). Drug-resistant influenza viruses used as controls were provided by the IRR. The sequences of detected viruses were analyzed to identify molecular markers of resistance to oseltamivir, zanamivir, and baloxavir marboxil (WHO, 2024b; Govorkova et al., 2022).

### 2.4 Whole-genome sequencing of influenza viruses

Influenza virus-positive samples with high viral loads (Ct values ≤ 28) were selected from patients across different age groups and geographical locations within the country. We subsequently performed whole-genome sequencing (WGS) of 48 influenza viruses, including 22 A(H1N1pdm09), 21 A(H3N2), and 5 B/Victoria strains. Sequencing was conducted at the NRL “Influenza and ARD” using the commercial Illumina Microbial Amplicon Prep Kit for Influenza A/B (Illumina, San Diego, CA, USA). Sequencing was performed using an Illumina MiSeq System with the 150-cycle v3 reagent kit (Illumina).

Additionally, another 59 samples were sequenced in the WHO Collaborating Center (London, UK). All sequences analyzed in this study are registered in the EpiFlu database of GISAID under the accession numbers listed in Supplementary Table S1.

### 2.5 Phylogenetic analysis

Sequences were retrieved from the GISAID EpiFlu for phylogenetic and molecular analyses. These included sequences of WHO-recommended vaccine strains, reference sequences with known subclade identities determined by the WHO, and sequences of viruses circulating in various European countries during the 2024–2025 season (ECDC, 2025b). Multiple sequence alignments were performed using the MUSCLE algorithm, which is a part of the Molecular Evolutionary Genetics Analysis software (version 11) (Tamura et al., 2021). Phylogenetic analyses were conducted using the maximum likelihood (ML) method along with the Hasegawa–Kishino–Yano (HKY) nucleotide substitution model with a gamma distribution (HKY + G) determined to be the best-fit model. The reliability of the tree topology was assessed using 1,000 bootstrap replicates.

The influenza virus clades and subclades were determined based on their distribution in the phylogenetic trees and clustering with reference strains. Clades and subclades were designated in accordance with the ECDC guidelines (ECDC, 2025b). Additionally, the Nextclade tool was used to verify the phylogenetic analysis results concerning the affiliation of the studied sequences with specific clades and subclades.^1^

### 2.6 Deduced amino acid sequence analysis and glycosylation prediction

The BioEdit software (version 7.2) was used to translate the full-length coding sequences of all viral segments into protein sequences. To identify amino acid substitutions, the sequences of all viral proteins were aligned and compared with those of egg-based vaccine strains recommended by WHO for the 2024–2025 season in the Northern Hemisphere (NH) (WHO, 2024c). Key amino acid substitutions in HA, defining distinct clades and subclades, were identified. Amino acid numbering of HA was performed after removal of the signal peptide.

Potential N-linked glycosylation sites on HA and NA were identified using NetNGlyc v.1.0, a tool from the Technical University of Denmark,^2^ with a threshold value of 0.5. N-linked glycosylation occurs when the amino acid sequence contains N–X–S/T (asparagine-X-serine/threonine), where X represents any amino acid except for aspartic acid or proline.

### 2.7 Statistical analysis

All statistical analyses were conducted using GraphPad Prism version 10.2.3 and SAS OnDemand for Academics (SAS^®^ Studio). Fisher’s exact test and Chi-square (*χ*^2^) tests were used for multiple comparisons of categorical variables, which are expressed as numbers with corresponding percentages. Statistical significance was set at *p <* 0.05. Continuous variables were reported as either means or medians, depending on the specific context of the analysis. To identify independent predictors of influenza virus detection, multivariable logistic regression analysis was performed. Separate multivariable logistic regression models were used to evaluate the associations between influenza virus type and the risks of hospitalization and pneumonia. Results were reported as odds ratios (ORs) and adjusted odds ratios (aORs) with 95% confidence intervals (CIs).

## 3 Results

### 3.1 Patient characteristics

Of the 2,981 enrolled patients, 917 (30.8%) were treated in primary healthcare facilities and 2,064 (69.2%) were hospitalized. The age distribution of the participants ranged from 10 days to 98 years, with a median age of 7 years (IQR 2–16 years) and a mean age of 19.0 years (SD 25.6). To examine infection patterns by age, the study population was divided into six distinct age groups: 0–4 (*n =* 1,112, 37.3%), 5–14 (*n =* 1,005, 33.7%), 15–29 (*n =* 220, 7.4%), 30–64 (*n =* 261, 8.8%), 65–79 (*n =* 282, 9.5%), and ≥ 80 (*n =* 94, 3.2%) years old. Age data were missing for seven (0.2%) patients. Among study participants of known sex, 1,490 (50.4%) were male, and 1,468 (49.6%) were female.

### 3.2 Viral detection

**Figure 1:**
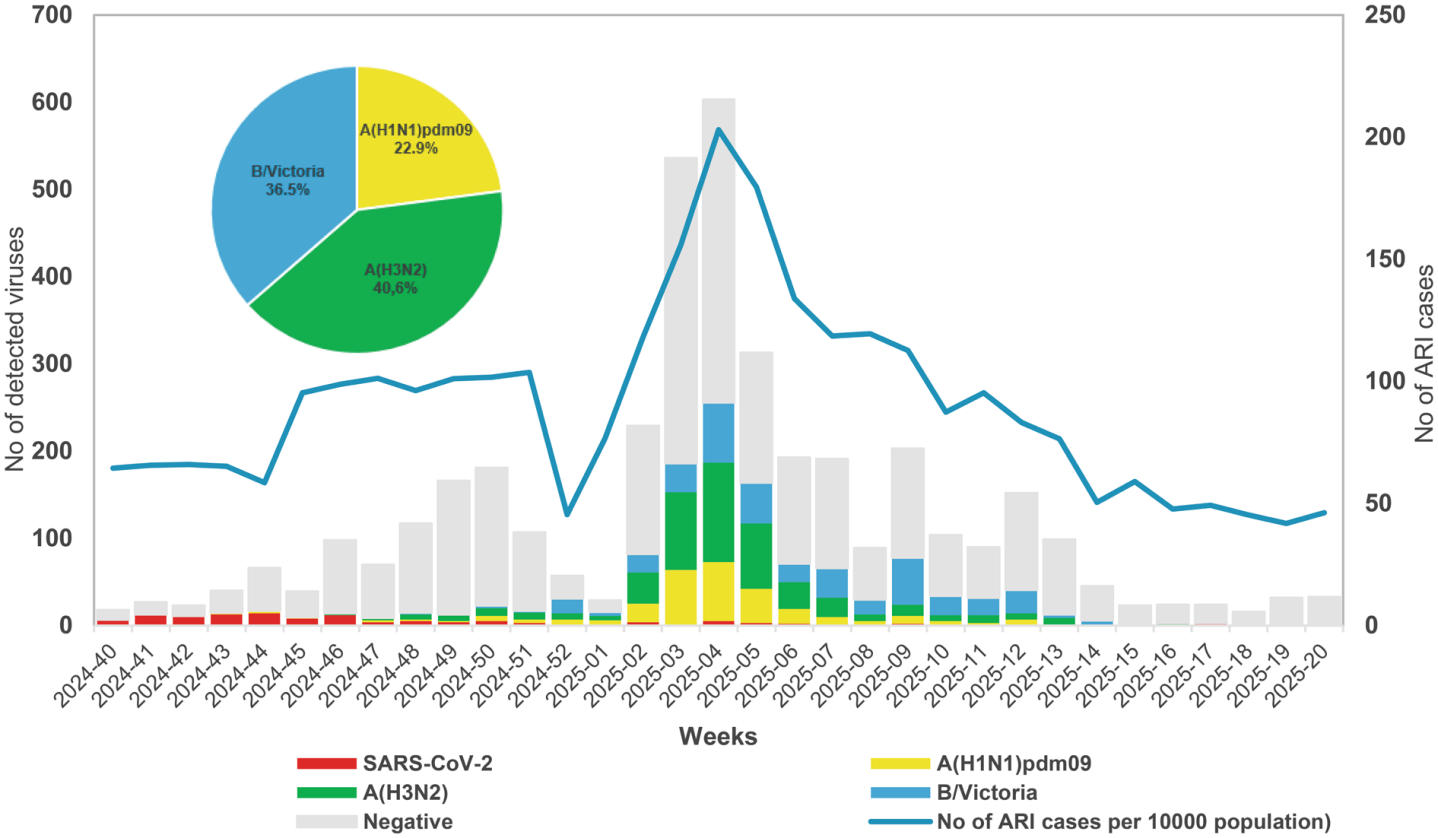
Weekly distribution of detected influenza viruses and SARS-CoV-2 during the 2024–2025 season.

To monitor respiratory virus circulation, 2,981 patients’ samples were screened for 13 respiratory viruses using real-time PCR. At least one respiratory virus was detected in 1,635 (54.8%) patients. Among the entire study population, 1,453 (48.7%) patients were infected with a single virus, 169 (5.7%) were co-infected with two viruses, 12 (0.4%) were co-infected with three viruses, and one (0.03%) was co-infected with four viruses. Influenza virus RNA was detected in 891 (29.9%) patient samples; influenza A and B viruses accounted for 63.5% (566 samples) and 36.5% (325 samples) of the positive cases, respectively. Of the influenza A-positive cases, 204 (36%) were identified as A(H1N1)pdm09 and 362 (64%) as A(H3N2). All detected influenza type B belonged to the Victoria lineage. Influenza virus activity began in week 48/2024, when the test positivity in primary care reached 10%, and peaked in week 4/2025. The highest influenza activity was observed in January 2025. Furthermore, the number of influenza type B-positive cases increased in March 2025. The last positive influenza case (H3N2) was in week 17/2025. SARS-CoV-2 exhibited greater activity in the fall of 2024; however, it almost disappeared from circulation with the emergence of influenza viruses (Figure 1).

SARS-CoV-2 was detected in 109 (3.7%) patient samples. In total, 833 patients were infected with non-influenza non-SARS-CoV-2 respiratory viruses. Of these, the largest proportion of positive patients had RVs (269; 9%), followed by BoV (182; 6.1%), RSV (178; 6%), AdV (92, 3.1%), hMPV (42, 1.4%), PIV-1 (40, 1.3%), PIV-2 (18, 0.6%), and PIV-3 (11, 0.4%).

**Figure 2:**
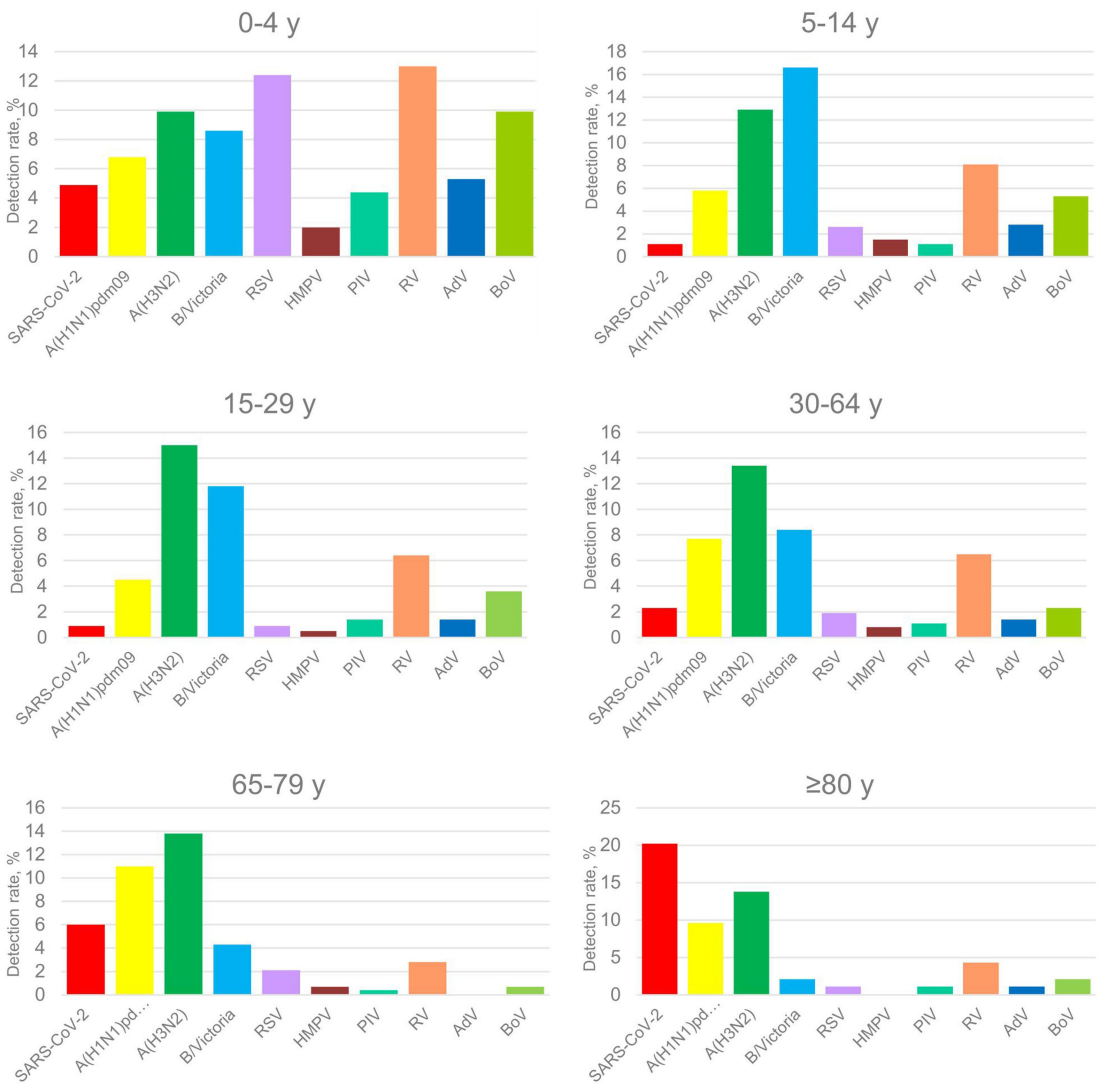
Positivity rates of respiratory viruses, detected in patients from different age groups (years) during the 2024–2025 season.

Among the 13 respiratory viruses studied, influenza A(H3N2) virus exhibited the highest positivity rate, followed by influenza B/Victoria and RV (*p <* 0.05). The smallest proportions were found for hMPV and PIV types 1/2/3 (<50 cases) (Figure 2).

### 3.3 Epidemiological characteristics

Age-dependent differences were observed in the incidence of respiratory viruses. The proportion of positive cases in the age groups 0–4, 5–14, 15–29, 30–64, 65–79, and ≥80 years was 65% (723/1112), 53.6% (539/1005), 43.6% (96/220), 42.9% (112/261), 40.4% (114/282), and 51.1% (48/94), respectively. Young children aged 0–4 years showed the highest detection rate, followed by children aged 5–14 years and the elderly (≥80 years).

The proportions of influenza virus-positive cases among age groups were 25.4% (282/1112), 35.3% (355/1005), 31.4% (69/220), 29.5% (77/261), 29.1% (82/282), and 25.5% (24/94) (Table 1). Influenza viruses most frequently affected children aged 5–14 years (35.3%), followed by those aged 15–29 years (31.4%). Influenza-positive individuals had a mean age of 18.7 years (SD 24.8, 1 month to 96 years) and a median age of 8 years (IQR 3–16), which was similar to influenza-negative patients (mean 19.1 years, SD 26.0; median 7 years; IQR 2–16). The predominant influenza A(H3N2) virus had the highest detection rate among 15–29-year-olds (15%), whereas A(H1N1)pdm09 was more prevalent (11%) in older adults (65–79 years old). Influenza B was most frequently detected in the age group 5–14 years (16.6%) (*p <* 0.05), and its detection rate decreased with age (2.1% in the elderly, ≥80 years). SARS-CoV-2 exhibited a unique detection pattern, with the highest positivity rate observed in the elderly (20%) and the lowest in 15–29-year-olds (0.9%) (*p <* 0.05). The average age of SARS-CoV-2-positive patients was 31.19 years (range, 1 month to 97 years; median, 4.5 years). Seasonal non-influenza and non-SARS-CoV-2 viruses were most frequently detected in the 0–4-year age group (47%), followed by 5–14-year-olds (21.3%). RV was the most prevalent seasonal non-influenza respiratory virus in the youngest age group, accounting for 13% (145/1112) of all cases, followed by RSV (12.4%, 138/1112). Of the influenza-positive patients, 414 (27.8%) were male, and 474 (32.3%) were female (*p <* 0.05). Female sex was independently associated with increased influenza virus detection (aOR 1.32, 95% CI 1.12–1.56).

**Table 1:** Comparison of socio-demographic and clinical characteristics according to influenza virus subtype among influenza positive-patients.

Variables	Total influenza positive (%)	A(H1N1) pdm09	A(H3N2)	B/Victoria lineage	*χ*^2^ (df), *p*-value	Total influenza infections		
						Single infections (%)	Double infection (%)	Triple infection (%)
Total patients (*n =* 2,981)	891 (29.9)	204 (6.8)	362 (12.1)	325 (10.9)		799 (26.8)	85 (2.9)	7 (0.2)
Sex								
Male (*n =* 1,490)	414 (27.8)	104 (7)	160 (10.7)	150 (10.1)	2.14 (2), 0.344	377 (25.3)	35 (2.3)	2 (0.1)
Female (*n =* 1,468)	474 (32.3)	100 (6.8)	200 (13.6)	174 (11.9)		419 (28.5)	50 (3.4)	5 (0.3)
Age (years)								
0–4 (*n =* 1,112)	282 (25.4)	76 (6.8)	110 (10)	96 (8.6)	56.01 (12), <0.0001	229 (20.6)	46 (4.1)	7 (0.6)
5–14 (*n =* 1,005)	355 (35.3)	58 (5.8)	130 (12.9)	167 (16.6)		328 (32.6)	27 (2.7)	0
15–29 (*n =* 220)	69 (31.4)	10 (4.5)	33 (15)	26 (11.8)		65 (29.5)	4 (1.8)	0
30–64 (*n =* 261)	77 (29.5)	20 (7.7)	35 (13.4)	22 (8.4)		73 (28)	4 (1.5)	0
65–79 (*n =* 282)	82 (29.1)	31 (11)	39 (13.8)	12 (4.3)		79 (28)	3 (1.1)	0
≥80 (*n =* 94)	24 (25.5)	9 (9.6)	13 (13.8)	2 (2.1)		23 (24.5)	1 (1.1)	0
without data (*n =* 7)	2	0	2	0		2	0	0
Clinical status								
Outpatients (*n =* 917)	253 (27.6)	50 (5.5)	124 (13.5)	79 (8.6)		231 (25.2)	21 (2.3)	1 (0.1)
Inpatients (*n =* 2064)	638 (30.9)	154 (7.5)	238 (11.5)	246 (11.9)	9.64 (2), 0.008	568 (27.5)	64 (3.1)	6 (0.3)
Tracheobronchitis (*n =* 155)	30 (19.4)	4 (2.6)	14 (9.0)	12 (7.7)		27 (17.4)	3 (1.9)	0
Bronchiolitis (*n =* 128)	18 (14.1)	3 (2.3)	7 (5.5)	8 (6.3)		11 (8.6)	7 (5.5)	0
Pneumonia (*n =* 433)	89 (20.6)	26 (6.0)	26 (6.0)	37 (8.5)	5.56 (2), 0.062	78 (18)	9 (2.1)	2 (0.5)
CNS involvement (*n =* 63)	9 (14.3)	0	5 (7.9)	4 (6.3)	3.81 (2), 0.149	9 (14.3)	0	0

Pearson *χ*^2^ test was used to compare categorical variables across influenza subtypes [A(H1N1)pdm09, A(H3N2), B/Victoria lineage]. Degrees of freedom (df) and *p*-values are shown. A *p* < 0.05 was considered statistically significant.

At least one respiratory virus was detected in 421 (45.9%) patients attending primary care centers and 1,214 (58.8%) hospitalized patients. Influenza infection was confirmed in 27.6% (253/917) of the outpatients and 30.9% (638/2064) of the inpatients. Multivariable logistic regression analysis indicated that hospitalized patients were more likely to be influenza-positive than those treated in primary care (aOR 1.33, 95% CI 1.10–1.61).

The detection rates in outpatients and inpatients were 5.5% (50/917) and 7.5% (154/2064) (*p <* 0.05) for influenza A(H1N1)pdm09 virus, 13.5% (124/917) and 11.5% (238/2064) for influenza A(H3N2) virus, and 8.6% (79/917) and 11.9% (246/2064) (*p <* 0.05) for influenza type B, respectively. SARS-CoV-2 was detected significantly more often in inpatients (4.9%) than in outpatients (0.8%) (*p <* 0.05). Furthermore, the incidence rates of RSV and AdV in hospitalized patients were higher than those in outpatients (*p <* 0.05) (Table 2).

**Table 2:** Number (%) of detected respiratory viruses among outpatients and inpatients.

Clinical status	Total tested	Total positive, *n* (%)	Influenza viruses, *n* (%)			SARS-CoV-2, *n* (%)	Seasonal non-influenza and non-SARS-CoV-2 respiratory viruses, *n* (%)							
			A(H1N1) pdm09	A(H3N2)	B/Vic		RSV	HMPV	PIV-1	PIV-2	PIV-3	RV	AdV	BoV
Outpatients	917 (30.8)	421 (45.9)	50 (5.5)	124 (13.5)	79 (8.6)	7 (0.8)	17 (1.9)	9 (1)	11 (1.2)	3 (0.3)	5 (0.5)	92 (10)	19 (2.1)	54 (5.9)
Inpatients	2064 (69.2)	1,214 (58.8)	154 (7.5)	238 (11.5)	246 (11.9)	102 (4.9)	161 (7.8)	33 (1.6)	29 (0.5)	15 (0.7)	6 (0.3)	177 (8.6)	73 (3.5)	128 (6.2)
Total	2,981	1,635	204	362	325	109	178	42	40	18	11	269	92	182

### 3.4 Viral detections in patients with different clinical diagnoses

**Figure 3:**
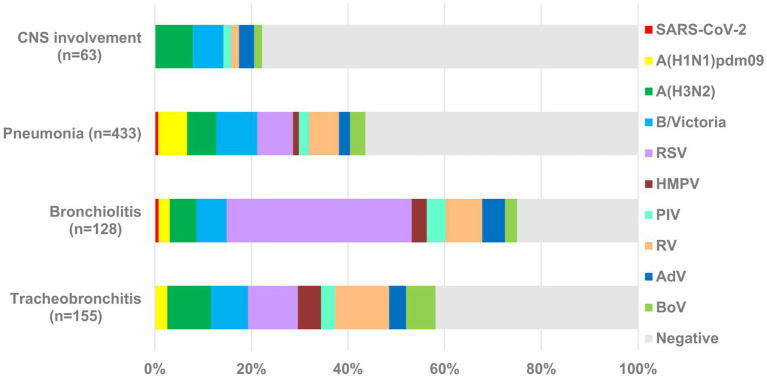
Proportions of respiratory viruses detected in patients with tracheobronchitis, bronchiolitis, pneumonia, and CNS involvement.

Viral respiratory infections typically manifest as upper respiratory tract symptoms but can also lead to complications in the lower respiratory tract, heart, muscles, and central nervous system (CNS). Therefore, we investigated the involvement of the studied respiratory viruses in the development of serious complications such as tracheobronchitis, bronchiolitis, pneumonia, and CNS-related symptoms (febrile seizures, cerebral edema, viral meningitis, and encephalopathy). Overall, 155 (5.2%) patients were diagnosed with tracheobronchitis, 128 (4.3%) with bronchiolitis, 433 (15%) with pneumonia, and 63 (2.1%) with CNS symptoms. Influenza infection was confirmed in 19.4% (30/155), 14.1% (18/128), 20.6% (89/433), and 14.3% (9/63) of patients with tracheobronchitis, bronchiolitis, pneumonia, and neurological complications, respectively. Non-influenza and non-SARS-CoV-2 viruses accounted for 45.2% (70/155), 76.6% (98/128), 28.4% (123/433), and 9.5% (6/63) of all cases, respectively. In contrast, SARS-CoV-2 was detected in markedly low proportions across the different diagnostic groups (0, 0.8, 0.7, and 0%) (Figure 3). The positivity rates for influenza A(H1N1)pdm09, A(H3N2), and type B viruses in patients with bronchiolitis were 2.3% (3/128), 5.5% (7/128), and 6.3% (8/128), respectively; those in patients with pneumonia were 6.0% (26/433), 6.0% (26/433), and 8.5% (37/433), respectively. Influenza A(H3N2) and B viruses were responsible for most CNS complications (*p <* 0.05); they were detected in five (7.9%) and four (6.3%) cases, respectively. Influenza type B was the most common causative agent among patients with pneumonia, accounting for 8.5% of all cases, followed by RSV (7.3%). Among children aged 0–4 years, pneumonia was attributed to influenza infections in 13.4% (24/177) and RSV infections in 12.4% (22/177). RSV was the predominant causative agent in children with bronchiolitis, accounting for 38.3% (49/128) of all cases. No deaths were reported for any patient.

Multivariable logistic regression analysis showed that compared to influenza A(H3N2), infection with A(H1N1)pdm09 was associated with a higher risk of hospitalization (aOR 1.52, 95% CI 1.01–2.30) and pneumonia (aOR 1.89, 95% CI 1.06–3.39). Additionally, infection with B/Victoria was also linked to increased odds of hospitalization (aOR 1.78, 95% CI 1.24–2.56), indicating clinically meaningful subtype-specific differences in disease severity.

### 3.5 Co-infections with respiratory viruses

**Figure 4:**
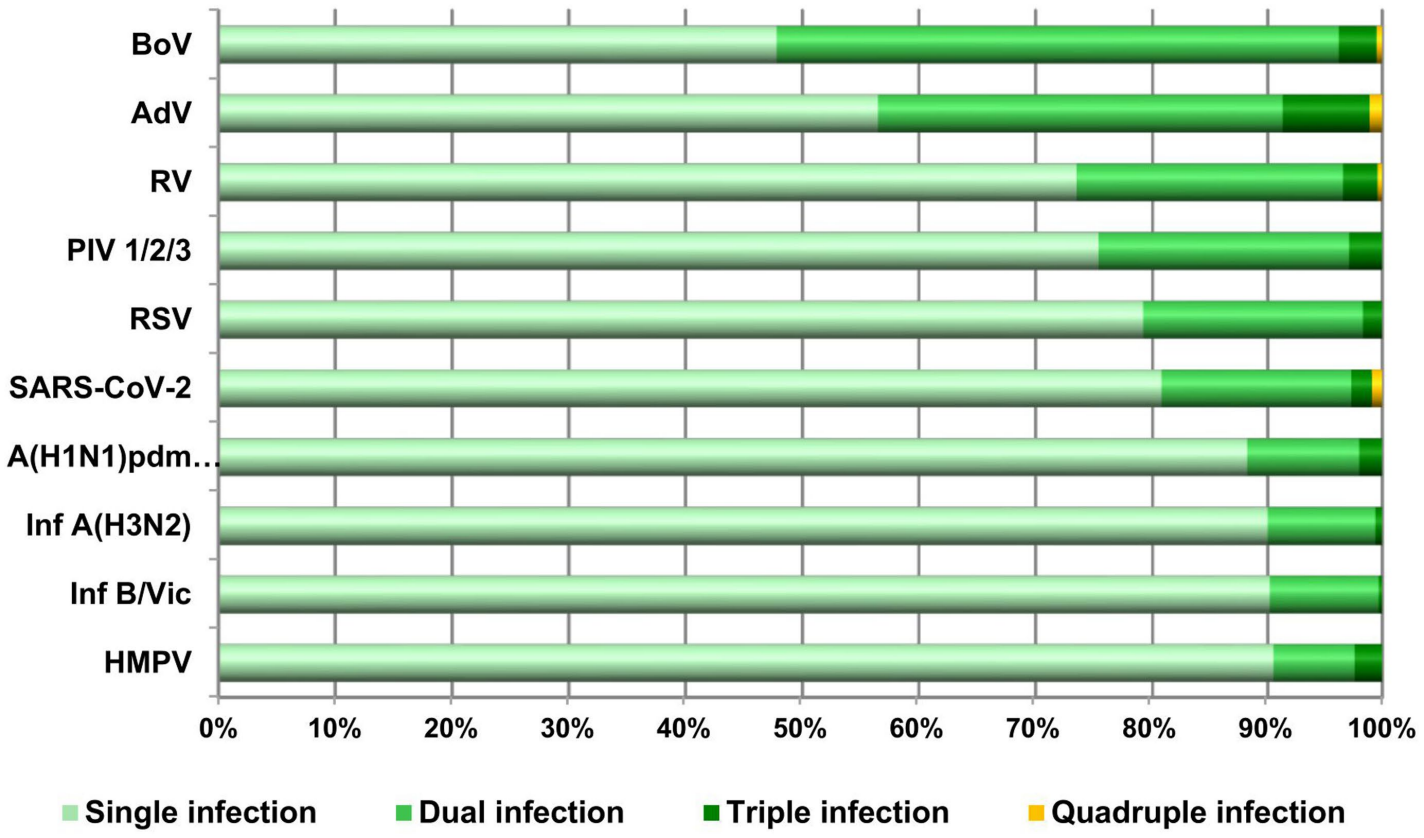
Proportions of single, dual, triple, and quadruple infections with participation of respiratory viruses.

In this study, more than one virus was detected in 182 (6.1%) patient samples. Of these, 92 (50.5%) samples showed co-detection of influenza viruses. Six cases of SARS-CoV-2 co-infection with influenza viruses were detected: A(H1N1)pdm09 (two cases), A(H3N2) viruses (two cases), and B/Victoria (two cases). The following seasonal non-influenza viruses were co-detected with influenza viruses: BoV (54 cases), RSV (13 cases), AdV (12 cases), RV (11 cases), and PIV2 (3 cases) (Table 3). Figure 4 shows the percentages of single, dual, triple, and quadruple infections of each respiratory virus. The influenza viruses B/Victoria, A(H3N2), and A(H1N1)pdm09, along with hMPV, were characterized by the lowest co-detection rates of 9.8% (32/325), 9.9% (36/362), 11.8% (24/204), and 9.5% (4/42), respectively. Moreover, they were not associated with quadruple infections. The viruses most commonly involved in mixed infections were BoV and AdVs, with co-detection rates of 52.2% (95/182) and 43.8% (40/92), respectively. SARS-CoV-2, RSV, PIV1/2/3, and RV occupied an intermediate position in terms of co-infection rates: 19.3% (21/109), 20.8% (37/178), 24.6% (17/69), and 26.4% (71/269), respectively. The most common cases of dual detection involved the following viral combinations: A(H3N2) and BoV (*n =* 24), RV and BoV (*n =* 24), and RV and AdV (*n =* 19) (Table 3).

**Table 3:** Number of single infections (in bold) and co-infections with participation of individual respiratory viruses.

Viruses	A(H1N1)	A(H3N2)	B/Vic	SARS-CoV-2	RSV	HMPV	PIV-1	PIV-2	PIV-3	RV	AdV	BoV
A(H1N1)	**180**			2	4					3	4	15
A(H3N2)		**325**		2	2			2		4	4	24
B/Vic			**292**	2	7			1		4	4	15
SARS-CoV-2				**88**	2		2	1		7	3	4
RSV					**141**					10	4	11
HMPV						**38**				3	1	1
PIV-1							**33**			4	1	1
PIV-2								**11**		2	2	
PIV-3									**8**	1	1	1
RV										**197**	19	24
AdV											**52**	6
BoV												**87**
Co-pathogen in dual inf.	20	34	31	18	34	3	6	6	3	62	32	88
Co-pathogen in triple inf.	4	2	1	2	3	1	1	1		8	7	6
Co-pathogen in quadriple inf.				1						1	1	1

Significant differences were observed in the frequency of co-infections across age groups. The percentage of cases with two or more viral infections was 11 and 8.3% in the youngest children and elderly individuals, respectively. In contrast, this percentage was substantially low (<4%) in the remaining age groups. Co-infections with three or four viral species were observed only in the youngest age group. The only case of quadruple infection was a 1-year-old child. Children under 4 years of age had the highest influenza virus co-detection rate (4.8%), followed by those aged 5–14 years (2.7%). Single-virus detection was predominant in the remaining age groups.

No significant difference in co-infection frequency was observed according to sex (*p* = 0.197). However, lower respiratory tract infection (LRTI) was more frequent among patients with co-infections (24.4%) compared to those with single infections (15.8%) [*χ*^2^(1) = 4.16, *p* = 0.041; Fisher’s exact *p* = 0.047]. Among patients with tracheobronchitis, 80 (51.6%) had single infections and 10 (6.5%) had double infections. Similarly, 76 (59.4%) children with acute bronchiolitis had mono-infections, whereas 19 (14.8%) had double and one (0.8%) had triple infections. Nineteen (4.5%) double, three (0.7%) triple, and 167 (38.6%) single infections were detected in patients with pneumonia. Only one case (1.6%) of co-infection was detected in a patient presenting with CNS symptoms. The rate of co-infection detection in patients with bronchiolitis was higher than that in the overall study population (*p <* 0.05). Influenza virus co-detection was observed in three, seven, and 11 patients with tracheobronchitis, bronchiolitis, and pneumonia, respectively (Table 1). RSV was predominantly detected as the sole pathogen in bronchiolitis cases, whereas influenza B/Victoria, A(H3N2), RSV, and RV were frequent causes of pneumonia in single infections Determination of antiviral susceptibility.

Thirty influenza virus isolates grown in cell cultures collected in Bulgaria during the 2024/2025 season underwent phenotypic analysis for resistance to the NA inhibitor oseltamivir. One B/Victoria strain showed a 3-fold increase in IC50 (0.67 nM) compared to the sensitive strains (median 0.2 nM).

### 3.6 Phylogenetic analysis of influenza viruses

**Figure 5:**
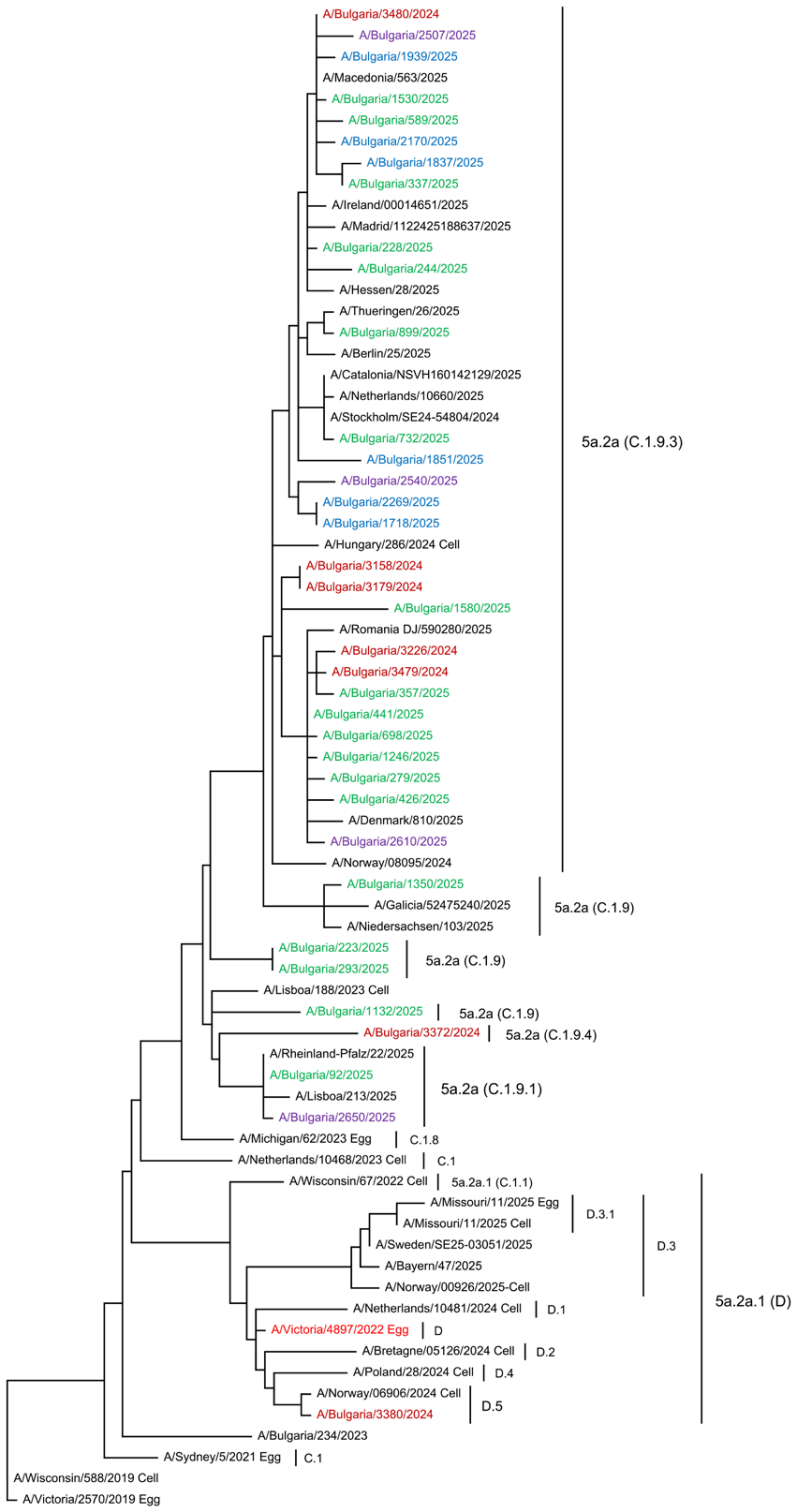
Phylogenetic analysis of the HA nucleotide sequences from influenza A(H1N1)pdm09 viruses circulating in Bulgaria during the 2024–2025 season. The phylogenetic tree was generated using the maximum likelihood method with 1,000 bootstrap replicates. The WHO-recommended vaccine strain is indicated in red. The source of each reference strain (egg or cell) is shown at the end of the designation. The Bulgarian strains, detected in December 2024 and in January, February, and March 2025, are shown in maroon, green, blue, and purple, respectively.

**Figure 6:**
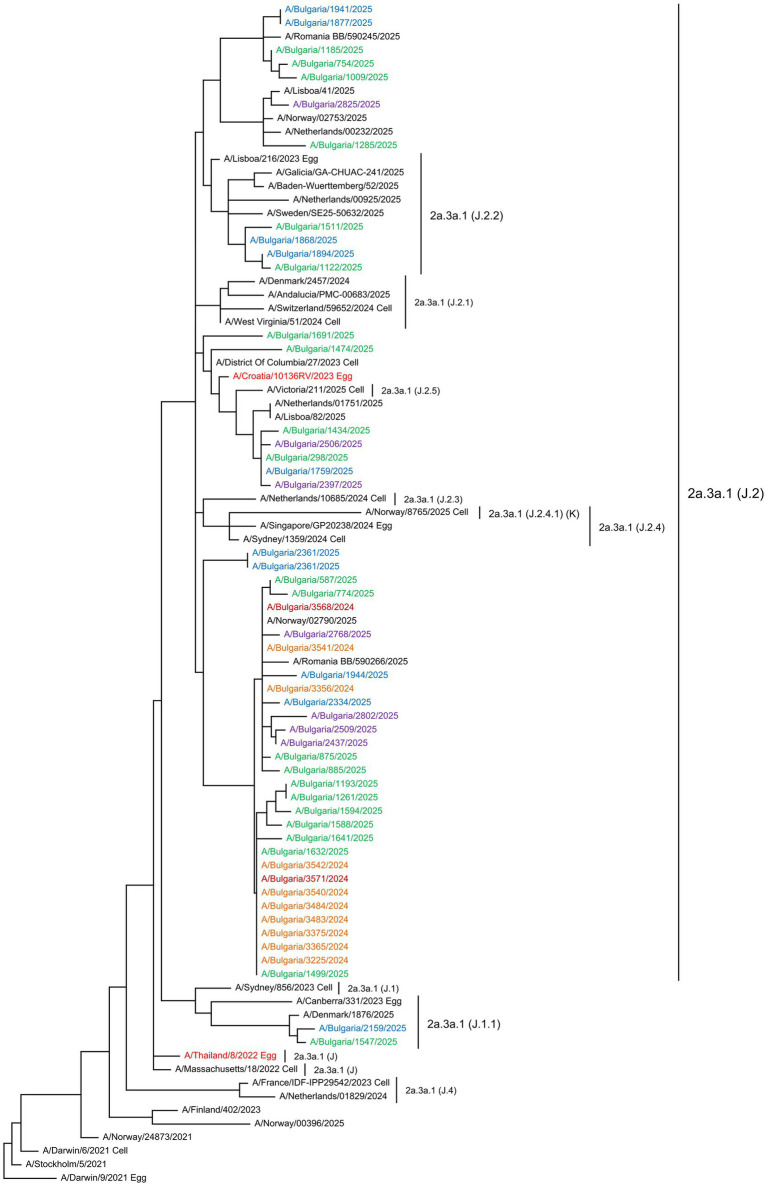
Phylogenetic analysis of the HA nucleotide sequences from influenza A(H3N2) viruses circulating in Bulgaria during the 2024–2025 season. The phylogenetic tree was generated using the maximum likelihood method with 1,000 bootstrap replicates. The WHO-recommended vaccine strains are indicated in red. The source of each reference strain (egg or cell) is shown at the end of the designation. The Bulgarian strains, detected in December 2024 and in January, February, and March 2025, are shown in maroon, green, blue, and purple, respectively.

**Figure 7:**
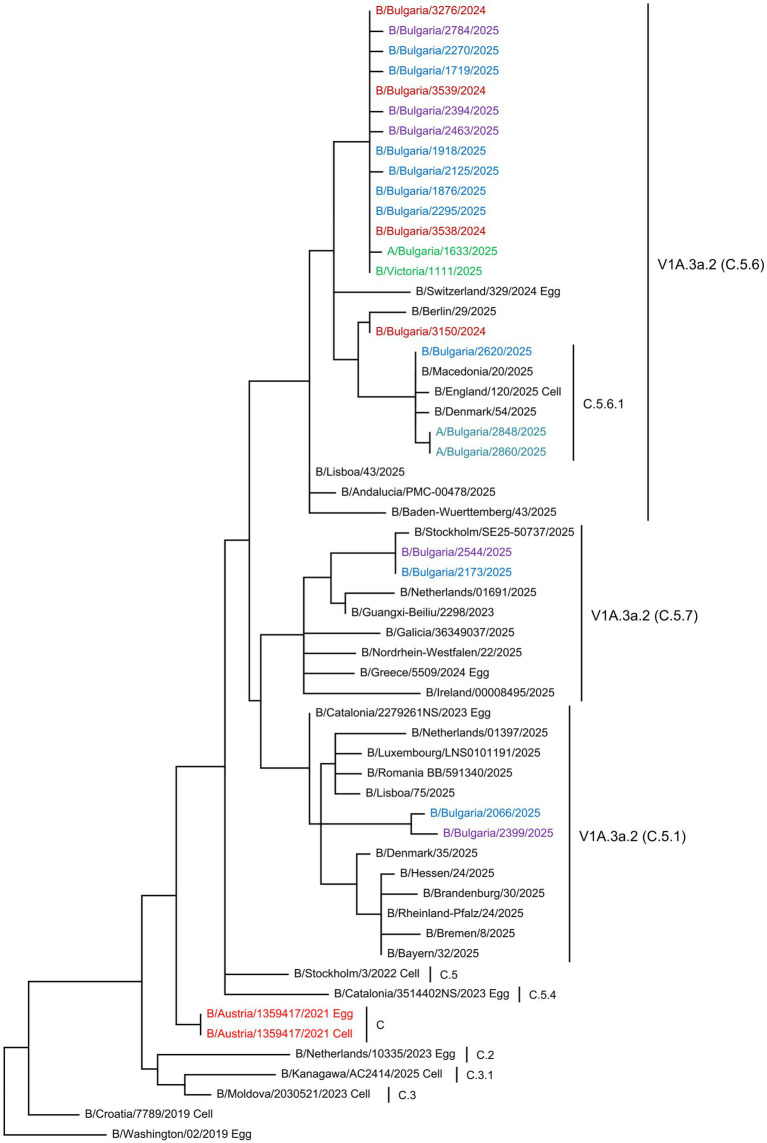
Phylogenetic analysis of the HA nucleotide sequences from influenza B/Victoria lineage viruses circulating in Bulgaria during the 2024–2025 season. The phylogenetic tree was generated using the maximum likelihood method with 1,000 bootstrap replicates. The WHO-recommended vaccine strains are indicated in red. The source of each reference strain (egg or cell) is shown at the end of the designation. The Bulgarian strains, detected in December 2024 and in January, February, and March 2025, are shown in maroon, green, blue, and purple, respectively.

Phylogenetic analyses of the HA gene sequences were conducted to determine the genetic relatedness of the Bulgarian influenza sequences to vaccine strains and globally circulating viruses (ECDC, 2025b). The phylogenetic trees included sequences of the studied strains, viruses circulating in other countries in the same period, reference viruses for individual clades/subclades, as determined by the WHO, and vaccine strains. The HA genes of the 36 Bulgarian A(H1N1)pdm09 strains clustered into two globally distributed genetic clades: 5a.2a (35 strains, 97.2%) and 5a.2a.1 (one strain, 2.8%). The second clade included the 2024–2025 NH vaccine strain A/Victoria/4897/2022. Viruses from the clade 5a.2a were further classified into four subclades: C.1.9 (four strains, 11.1%), C.1.9.1 (two strains, 5.6%), C.1.9.3 (28 strains, 77.8%), and C.1.9.4 (one strain, 2.8%). The only representative of clade 5a.2a.1 belonged to subclade D.5 (Figure 5). Based on the HA gene classification, the 49 studied A(H3N2) viruses belonged to three subclades within clade 2a.3a.1: J.2, which accounted for most of the viruses (43 strains, 76.8%); J.2.2 (four strains, 7.1%); and J.1.1 (two strains, 3.6%). No representatives of subclade J.2.4.1 (K) were identified among the Bulgarian strains (Figure 6). All 22 B/Victoria-lineage viruses detected in Bulgaria belonged to the genetic clade V1A.3a.2. These aligned with B/Austria/1359417/2021, the vaccine strain for the 2024–2025 NH influenza season. The study strains were assigned to subclade C.5 and further categorized into three subclades: C.5.1 (two strains, 9.1%), C.5.6 (18 strains, 81.8%), and C.5.7 (two strains, 9.1%). Three C.5.6 strains were clustered into separate subclades (C.5.6.1) (Figure 7).

### 3.7 Analysis of amino acid substitutions

The surface glycoproteins HA and NA are targets of neutralizing antibodies and have rapidly evolved to evade immune pressure (Blackburne et al., 2008). Therefore, we analyzed the amino acid substitutions in these glycoproteins to elucidate the mechanisms underlying antigenic drift, which is responsible for the limited effectiveness and narrow breadth of influenza vaccines. The complete HA and NA amino acid sequences of Bulgarian influenza viruses were compared with those of WHO NH egg-based vaccine strains to identify substitutions that might affect vaccine effectiveness.

#### 3.7.1 A(H1N1)pdm09

The HA and NA sequences of 36 Bulgarian A(H1N1)pdm09 isolates were compared with those of the vaccine strain A/Victoria/4897/2022 and representatives of particular genetic clades/subclades. The studied viruses showed high nucleotide homology of the HA gene compared to the vaccine virus (96.996–99.470%) (FluSurvey Tool, 2025). In total, 22 amino acid changes were detected compared to the vaccine strain: 17 in the HA1 domain (globular head) and five in HA2 (stem region). The HA1 R223Q substitution was fixed and present in viruses from all genetic groups. Eight substitutions were observed in all strains within clade 5a.2a (subclades C.1.9, C.1.9.1, C.1.9.3, and C.1.9.4), with a frequency of 97.2%. The substitution S137P appeared in 29 (80.6%) strains from these subclades, except for C.1.9.1. The substitution I510T was detected in both subclades C.1.9 and C.1.9.3, with a frequency of 80.6%. The other seven substitutions were observed in single subclades. Among the epitope-specific substitutions, three (I166V, K169Q, and R205K), two (S137P and R142K), and one (N125D) affected the epitopes Ca1, Ca2, Sa, respectively (Wilson et al., 2015). Mutations R142K (in Ca2) and K169R (in Ca1) were detected in 97.2% of the strains, whereas S137P was observed in 80.6% of the strains. Only one virus harbored a substitution in the strain-specific epitope Sa. No variations were identified in the receptor-binding site (RBS) 190-helix (residues 184–191) (Sriwilaijaroen and Suzuki, 2012). All substitutions are extensively described in Supplementary Table S2. The Bulgarian viruses harbored eight conserved putative N-glycosylation motifs in HA (HA1 positions 10, 23, 87, 162, 276, and 287, and HA2 positions 154 and 213). Although all these motifs were present in the vaccine strain, a small alteration was observed: the A277T substitution altered the N-glycosylation motif NAT at position 276 to NTT.

Substitutions at 13 positions in the NA of Bulgarian A(H1N1)pdm09 viruses were identified and compared with those in the vaccine strain. Substitutions D50N, S200N, and E382G were present in all strains, and I264T appeared in four subclades at a frequency of 94.4%. The other two substitutions, S52N and I46V, also exhibited high frequency (80.6 and 61.1%, respectively). The remaining seven substitutions were observed in single subclades (Supplementary Table S3). The mutation S200N was related to antigenic drift (FluSurvey Tool, 2025). Eight N-glycosylation motifs at positions 42, 50, 58, 63, 68, 88, 146, and 235 of NA were observed in Bulgarian viruses. The substitution S52N resulted in the loss of potential N-glycosylation sites in 29 (80.6%) strains. Contrastingly, the H275Y substitution that confers resistance to oseltamivir and peramivir was not detected in the NA of the viruses studied (Hussain et al., 2025). The mutation S247N, associated with resistance to NA inhibitors in recent A(H1N1)pdm09 strains, was also not detected (Saubi et al., 2025). The mutations I264T and S153N, identified in 34 and one study strains, respectively, are related to mild drug resistance (FluSurvey Tool, 2025).

We compared the sequences of the internal proteins PB2, PB1, PA, NP, MP, and NS of influenza A(H1N1)pdm09 viruses with those of the vaccine strain. Several amino acid substitutions were detected: five in PB2, two in PB1, six each in PA and NP, and one in NS1. M1 had no substitutions. (Supplementary Table S4). Importantly, no amino acid changes were observed at the baloxavir-binding site (positions 20, 24, 34, 37, and 38) (Govorkova et al., 2022).

#### 3.7.2 A(H3N2)

Compared to the NH 2024–2025 egg-based vaccine strain A/Thailand/8/2022, Bulgarian A(H3N2) viruses exhibited nucleotide homology of the HA gene ranging from 98.410 to 99.117% (FluSurvey Tool, 2025). All 49 analyzed A(H3N2) strains belonged to clade 2a.3a.1 and demonstrated amino acid changes at 18 positions of the HA protein relative to the vaccine virus. The Y195F substitution was detected in all viruses from the three subclades. Viruses from subclades J.1.1, J.2, and J.2.2 exhibited additional specific amino acid substitutions, with four in J.1.1, 12 in J.2, and five in J.2.2. The substitutions N122D, I182V, and K276E were present in both subclades J.2 and J.2.2, occurring at a frequency of 95.9%. Additionally, S145N was detected in 13 strains (26.5%) across the three subclades. The remaining 13 substitutions were unique to individual subclades.

Compared to the corresponding vaccine strain, eight epitope-specific substitutions were identified across four antigenic regions: A (five substitutions), C (one substitution), D (one substitution), and E (one substitution) (Stray and Pittman, 2012). Twelve potential N-glycosylation sites were detected, 11 of which were located in the HA1 domain at positions 8, 22, 38, 45, 63, 96, 122, 126, 133, 165, 246, and 285. One site was detected in the HA2 domain at position 154. However, these sites were not present in all studied strains. Substitutions N8D, T65K, N94K, S96I, N122D, S124N, and T135A/K resulted in the loss of potential N-glycosylation sites (-CHO) in 2, 4, 1, 1, 47, 4, and 10 strains, respectively.

Amino acid polymorphism was identified in 11 positions of the NA protein compared with the A/Thailand/8/2022 vaccine strains. Substitution K210R was present in all strains, and substitution R400K appeared in all three subclades (J.1.1, J.2, and J.2.2), at a frequency of 42.6%. The remaining nine substitutions were specific to individual subclades: two in J.1.1, five in J.2, and two in J.2.2. Similar to the vaccine strain, the Bulgarian NA sequences harbored nine potential N-glycosylation motifs at positions 61, 70, 86, 146, 200, 234, 245, 367, and 463. Two motifs (146 and 367) were located near the active enzymatic site (de Graaf and Fouchier, 2014). Moreover, no substitutions affecting sensitivity to NA inhibitors were detected in the NA protein of the analyzed A(H3N2) viruses.

A few amino acid substitutions were detected in the remaining viral proteins: PB2 (four), PB1 (zero), PA (five), NP (four), M1 (one), and NS1 (seven). NS1 and NP showed higher variability, whereas PB1 and M1 exhibited greater genetic stability (Bian et al., 2025). Substitutions associated with decreased baloxavir activity (at positions 38, 34, and 28 of PA) were not observed (Govorkova et al., 2022).

#### 3.7.3 B/Victoria

All 22 Bulgarian B/Victoria lineage viruses belonged to clade V1A.3a.2 (C) and subclades C.5.1, C.5.6, C.5.6.1, and C.5.7. The HA gene in the viruses studied was highly homologous to that of the vaccine strain B/Austria/1358417/2021 (99.141–99.485% homology) (FluSurvey Tool, 2025). The HA protein sequences harbored amino acid substitutions at eight positions compared to the vaccine strain. D194E was detected in all the study strains. D129N and T196A were detected in subclades C.5.6 and C.5.6.1, whereas E180K was observed in subclades C.5.1, and C.5.7. Substitutions E128K, D129N, and E128G define subclades C.5.1, C.5.6, and C.5.7, respectively, and were detected in the relevant Bulgarian strains. The new subclade C.5.6.1 was characterized by additional substitutions T37I and E128D (ECDC, 2025b).

Influenza B viruses contain four major antigenic regions in the HA1 domain: 120 loop (116—137), 150 loop (141–150), 160 loop (162–167), and 190 helix (194–202) (Wang et al., 2008). Substitutions E120K, E128K/G/D, and D129N affected the 120-loop, whereas D194E and T196A affected the 190-helix. No amino acid substitutions were observed in the remaining antigenic epitopes (150-loop and 160-loop) (Wang et al., 2007). Eleven putative N-glycosylation motifs were detected: seven in the HA1 domain at positions 25, 59, 145, 163, 230, 301, and 330, and four in the HA2 domain at positions 145, 171, 184, and 216. N-glycosylation motifs 145 and 163 belonged to the 150-loop and 160-loop antigenic sites, respectively.

The B/Victoria lineage viruses harbored 11 amino acid substitutions in the NA protein compared to the vaccine strains; I459V was present in all strains. Substitution G378E was identified in both subclades C.5.1 and C.5.7, whereas S397N and K404R were present in C.5.6 and C.5.6.1. Additional amino acid substitutions were detected: two in subclade C.5.1, three in subclade C.5.6, and one in subclade C.5.7. Substitutions I459V and K360N are associated with mild drug resistance (FluSurvey Tool, 2025). Four potential N-glycosylation motifs were detected at positions 56, 64, 144, and 284.

Significantly fewer substitutions were detected in the internal viral proteins PB2 (one), PB1 (four), PA (two), NP (two), M1 (two), and NS1 (three).

## 4 Discussion

This study examined the circulating patterns of influenza viruses detected in Bulgaria during the 2024–2025 season, along with those of other respiratory viruses reported in the country at the same time. Of the various respiratory viruses identified, influenza A(H3N2) and the B/Victoria lineage were the predominant causes of ARI, whereas SARS-CoV-2 circulated at a markedly low level. Contrary to our findings, ECDC data indicated that A(H1N1)pdm09 was the most prevalent influenza virus (ECDC, 2025b), which may be explained by differences in the geographical distribution of individual influenza virus subtypes. Type B viruses had relatively high activity, and all identified strains belonged to the B/Victoria lineage. Influenza virus circulation exhibits seasonal patterns (Uyeki et al., 2022). In our study, the seasonality of influenza circulation closely resembled patterns observed in other countries with temperate climates in the Northern Hemisphere. As in previous seasons, it was characterized by typical peaks between December and February, along with a smaller wave of influenza B at the end of the season (Korsun et al., 2023). Understanding the seasonal patterns of influenza viruses can help to determine the optimal timing for vaccine administration and identify periods of increased healthcare needs.

Our findings indicate that susceptibility to viral respiratory infections varies significantly by age group. Young children aged 0–4 years were the most frequently affected by respiratory viruses, followed by children aged 5–14 years and the elderly. In line with our previous study, the highest proportion of influenza virus-positive cases was observed among children aged 5–14 years and in young adults aged 15–29 years (Korsun et al., 2023). Conversely, SARS-CoV-2 was most prevalent among the elderly, consistent with the observations reported in other studies (Ruivo et al., 2025). The high influenza prevalence in children aged 5–14 years likely reflects school-related exposure combined with suboptimal vaccination coverage in this age group. In Bulgaria, influenza vaccination coverage is low, around 7% for the entire eligible population (Immunizations in 2025, 2025). These findings highlight the importance of age-specific approaches in managing respiratory infections and demonstrate the need for stronger recommendations for seasonal influenza vaccination in pediatric populations.

The detection rates of influenza A(H1N1)pdm09 and B/Victoria were higher in inpatients than in outpatients (*p <* 0.05). Significant differences in infection rates were observed between outpatients and hospitalized patients for SARS-CoV-2, RSV, and AdVs, which is consistent with previous studies (Kelly et al., 2025). Our findings suggest that these viruses are often associated with the need for hospitalization.

This study demonstrated the causative role of influenza viruses in pneumonia and CNS complications, confirming their strong association with disease severity. Severity in this surveillance dataset was assessed using recorded clinical diagnoses (e.g., LRTI and CNS involvement) rather than standardized severity scores or outcome measures such as ICU admission or length of stay. Our results are consistent with recent epidemiological surveillance data indicating that influenza A/B, RSV, and SARS-CoV-2 are the predominant causative agents of community-acquired pneumonia (Contes and Liu, 2025). Although neurological complications associated with influenza, such as febrile seizures and encephalopathy, are uncommon, they were more frequently linked to influenza A(H3N2) and B/Victoria viruses. These findings highlight the importance of conducting influenza tests at early illness onset in patients presenting with fever and neurological symptoms during the influenza season. The early consideration of antiviral therapies is also recommended. Additionally, the data support vaccination as a crucial preventive measure against severe outcomes, including pneumonia and CNS complications. Although influenza viruses have been detected in cases of bronchiolitis, RSV remains the predominant clinically significant pathogen.

The increased use of multiplex molecular methods in diagnostic virology has facilitated the simultaneous detection of multiple respiratory viruses in the same clinical specimen. The prevalence of respiratory virus co-infections varies significantly across studies worldwide, owing to factors such as geographical location, seasonality, patient age, social and immune status, diagnostic capabilities, and the specific viruses involved (Babawale and Guerrero-Plata, 2024). Goka et al. (2015) found that the frequency of viral co-infections varied by 5–62% in their systematic review, with an average of 23% (Goka et al., 2015). In our study, 6.1% of the patients were infected with more than one virus, with the highest rate of multi-pathogen detection observed in children aged 0–4 years (11.1%) and the lowest rate in patients aged 65–79 years (1.4%). Our co-infection rate was consistent with findings from recent studies in Italy (5.7%) (Liotti et al., 2025) and Brazil (7.5%) (Siqueira et al., 2024), but was significantly lower than that reported in India (14.9%) (Deval et al., 2024). Studies have reported different predispositions to respiratory virus co-infections in individuals of different age groups (Mandelia et al., 2021). Most researchers reported the highest co-infection rate in young children, which can be explained by their underdeveloped immune systems and increased opportunities for simultaneous exposure in childcare facilities (Kelly et al., 2025; Tomita et al., 2025). The high rate of mixed viral infections highlights the vulnerability of this age group and emphasizes the need to implement age-specific infection control measures.

Data from different countries show differences in how often individual viruses are involved in co-infections. In our study, influenza viruses had a low co-detection rate, whereas BoV and AdV were the most common co-infecting viruses. In contrast, other studies identified influenza viruses and RVs as the most common co-infectious pathogens (Liotti et al., 2025; Han et al., 2025). The low incidence of co-infections involving influenza viruses indicates that these viruses more often act as primary, clinically significant pathogens.

In this study, mixed infections were more prevalent in inpatients than in outpatients (*p <* 0.05). Similar trends were observed by Petat et al. (2024), who reported that the risk of viral co-detection in hospitalized infants was 4.1 times higher for RSV and 93.9 times higher for RV than that in primary care settings (*p <* 0.001) (Petat et al., 2024). In the present study, the rate of co-infection among patients with bronchiolitis was higher than that in the overall study population (*p <* 0.05). Consistent with our results, researchers from Italy reported a higher prevalence of co-infections in children with lower respiratory tract infections than in those with upper respiratory tract infections (Di Maio et al., 2024). The higher prevalence of mixed infections in hospitalized patients may reflect increased testing intensity and underlying vulnerability, and warrants careful interpretation in surveillance datasets, but nonetheless underlines the importance of early diagnosis and targeted treatment. These findings suggest that some viruses interact synergistically to exacerbate disease severity. However, the clinical significance of mixed infections is yet to be definitively established. Co-infections between SARS-CoV-2 and influenza strains are associated with more severe disease forms than single infections (Contes and Liu, 2025). These viruses can induce strong inflammatory responses and cytokine storms, which greatly complicate their clinical course. In our study, six cases of coinfection with SARS-CoV-2 and influenza viruses were identified in hospitalized patients. Vaccination against these pathogens can provide effective protection against severe diseases and fatal outcomes. Researchers have expressed conflicting opinions regarding the impact of respiratory viral co-infections on disease severity and outcomes. Some studies have found a strong correlation between co-infection, disease progression, and worsening of symptoms (Siqueira et al., 2024; Li et al., 2020). Brazilian authors reported that co-infections involving influenza viruses are significantly associated with cardiopathy and death (Gregianini et al., 2019). However, other researchers reported no correlation between co-infection and worsened clinical outcomes (Scotta et al., 2016; Asner et al., 2014). Thus, factors such as virus–virus interactions, individual characteristics of host immune responses, and pathogenicity of the involved viruses must be considered to explain the association between co-infection and disease severity (Tomita et al., 2025). Importantly, early diagnosis of viral co-infection may prevent unnecessary antibiotic use, improve patient treatment, and reduce the risk of severe complications.

The detection of multiple viruses in a single sample can be attributed to the high sensitivity of PCR assays. These assays can detect substantially small amounts of viral nucleic acids that may be present during the incubation period or convalescence phase of the disease. Prolonged viral shedding following a previous infection or silent circulation of some viruses may also be responsible (Reller et al., 2024). For example, BoV DNA can persist in airway secretions for up to 4.5 months after an acute infection in hospitalized patients, which may explain its frequent involvement in cases of co-infections (Blessing et al., 2009).

Mutations in the surface proteins HA and NA limit the efficacy of influenza vaccines. To elucidate the mechanisms by which these viruses evade immune responses, comprehensive phylogenetic and molecular analyses of their genes and proteins were performed. Phylogenetic analysis revealed that Bulgarian A(H1N1)pdm09 viruses belonged to several genetic subclades: C.1.9, C.1.9.1, C.1.9.3, and C.1.9.4 within clade 5a.2a; and D5 within clade 5a.2a.1. Only one strain clustered with the 2024–2025 NH vaccine strain A/Victoria/4897/2022 within clade 5a.2a.1. Despite genetic differences, circulating viruses from the 5a.2a clade remain antigenically close to the current vaccine strain, according to WHO data (ECDC, 2025b). In total, 22 amino acid changes were identified compared to the vaccine strain, including six substitutions at the HA antigenic sites: Ca1 (three), Ca2 (two), and Sa (one) (Wilson et al., 2015). Three substitutions located at the constant Ca1 (97.2%) and Ca2 (80.6%) sites were present at high frequencies. No changes were observed in epitope Sb or the RBS. The specific epitopes Sa and Sb, located at the top of the HA protein, and the constant epitopes Ca1, Ca2, and Cb, situated near the stalk domain, play crucial roles in immune evasion (Martínez et al., 2024). Previous research showed that substitutions involving more than four amino acids in HA antigenic sites and located at two or more antigenic sites may lead to antigenic drift (Wilson and Cox, 1990). Koel et al. (2025) reported that substitutions in or near the RBS may alter the antigenic properties and adaptability of the virus, thereby enhancing transmissibility.

Bulgarian influenza A(H3N2) viruses belonged to three globally circulating genetic subclades: J.1.1, J.2, and J.2.2, all within clade 2a.3a.1. No representatives of subclade J.2.4.1 (K) were detected among the examined sequences. This subclade, with reference strain A/Norway/8765/2025, shows significant divergence from the NH A(H3N2) vaccine strain and has become globally widespread since May 2025 (Kirsebom et al., 2025).

Bulgarian A(H3H2) sequences harbored 18 amino acid changes compared to the corresponding vaccine strain, including eight substitutions at antigenic sites A (five substitutions), C (one substitution), D (one substitution), and E (one substitution). Specifically, antigenic site A accumulated the highest number of substitutions. Substitutions at antigenic sites A and B, located at the top of HA flanking the RBS, are linked to transmission and immune evasion (Chambers et al., 2015; Popova et al., 2012). Key HA antigenic positions 144, located at site A, and 155, 156, 158, 159, 189, and 193, located at site B near the RBS, were responsible for major antigenic changes in A(H3N2) viruses between 1968 and 2003 (Koel et al., 2013). In the present study, no variations were observed at these positions. A(H3N2) viruses exhibited high levels of amino acid polymorphisms in HA antigenic regions and a higher degree of glycosylation than the A(H1N1)pdm09 and B/Victoria lineages. A notable feature of this subtype is its rapid evolution, which has led to reduced vaccine effectiveness and the need for more frequent updates of this vaccine component (Bian et al., 2025; Bedford et al., 2014).

The Bulgarian B/Victoria lineage viruses analyzed belonged to the four globally circulating subclades (Peng et al., 2025). The HA protein sequences harbored eight amino acid substitutions compared to the vaccine strain. Of these, two affected the antigenic site 120-loop, whereas one was located in the 190-helix. These findings revealed that more epitope-specific substitutions in the HA protein were concentrated in the antigenic 120-loop. The 120-loop exhibits the highest variability, and amino acid substitutions in this region may affect antigenicity (Lugovtsev et al., 2007; Chen and Holmes, 2008). Our results are consistent with those of earlier studies that reported lower variability and slower evolutionary rates of type B viruses (Vijaykrishna et al., 2015; Wang et al., 2024).

Glycosylation of surface proteins is an important strategy for immune evasion. The present study detected several variations in N-glycosylation sites compared to vaccine strains. Changes in N-linked glycosylation can affect the antigenicity of these viruses and their sensitivity to neutralizing antibodies (Kim et al., 2018).

Importantly, no influenza viruses were identified to have reduced susceptibility to oseltamivir. WHO classifies this as a 10- to 100-fold increase in the IC50 for influenza A and a 5-to 50-fold increase for influenza B (Hussain et al., 2025). Only a few genetic substitutions linked to decreased susceptibility to the NA inhibitor oseltamivir were identified in our study. Globally, low frequencies (0.1–0.2%) of seasonal influenza viruses exhibiting reduced or highly reduced inhibition by NA inhibitors have been observed (Hussain et al., 2025). These findings indicate that antiviral drugs remain effective against circulating strains.

Our study had several limitations. First, we provide a more detailed analysis of the epidemiological characteristics of influenza infections than those of infections caused by other respiratory viruses. In addition, most study participants were children aged 0–4 years, followed by those aged 5–14 years. Representation of other age groups, particularly individuals aged ≥80 years, was limited. Therefore, future studies should include a higher number of patients in underrepresented age groups. In this study, we evaluated associations between influenza co-infection and several diagnosis-based severity proxies (hospitalization, pneumonia, LRTI, CNS involvement); however, we lacked standardized severity outcomes (ICU admission, ventilation, length of stay) and therefore could not assess severity gradients or clinical outcomes in detail. The study was prospective, observational, and based on molecular detection of viral nucleic acids. As a result, it cannot ascertain the temporal sequence of infections, active viral replication, or the role of each virus in disease severity. Although we identified several mutations in viral proteins, we did not assess their association with disease severity. Despite these limitations, our study offers valuable insights into the range of respiratory viral pathogens circulating in Bulgaria, as well as the genomic diversity and evolution of influenza viruses during the 2024–2025 season. Thus, our findings may be crucial for the development of public health strategies aimed at reducing the burden of ARIs.

In conclusion, our study demonstrated high influenza virus activity in Bulgaria during the 2024–2025 season, as well as its association with serious clinical complications. The detection of multiple viral pathogens in patients with ARIs underscores the importance of multiplex diagnostic assays, as co-infections can significantly affect the etiological diagnosis, clinical presentation, treatment outcomes, vaccination strategies, and infection control measures. The relatively infrequent involvement of influenza viruses in co-infections indicates that they can independently cause severe respiratory illnesses. Our study also revealed substantial genetic diversity among circulating influenza viruses in Bulgaria, providing important insights into their evolutionary dynamics and immune evasion. The concurrent circulation of multiple subclades suggests several independent introductions of these viruses. In addition, all genomic segments of influenza viruses are prone to mutations, particularly those encoding surface glycoproteins. This finding emphasizes the need for ongoing and enhanced surveillance to better understand influenza virus evolution and assess the effectiveness of public health strategies aimed at controlling the virus.

## Data Availability

The datasets presented in this study can be found in online repositories. The names of the repository/repositories and accession number(s) can be found in the article/supplementary material.

## Footnotes

1
https://nextstrain.org/

2
https://services.healthtech.dtu.dk/service.php?NetNGlyc-1.0
