# Supplementary material for: Viral pathogens in the etiology of acute respiratory infections in Bulgaria during the 2024–2025 season and genetic diversity of circulating influenza viruses
Source: Front Microbiol. 2026 Apr 16;17:1785399. doi: 10.3389/fmicb.2026.1785399 (PMC13131023; doi:10.3389/fmicb.2026.1785399)
Supplement: Supplementary file 1 [file Table_1.docx]

**Table S1**. GISAID virus/sequence identification/accession numbers of Bulgarian influenza strains analyzed in this study, along with the vaccine strains

| **A(H1N1)pdm09 strains**  *A/Victoria/4897/2022*  A/Bulgaria/1132/2025  A/Bulgaria/1246/2025  A/Bulgaria/1350/2025  A/Bulgaria/1530/2025  A/Bulgaria/1580/2025  A/Bulgaria/1718/2025  A/Bulgaria/1837/2025  A/Bulgaria/1851/2025  A/Bulgaria/1939/2025  A/Bulgaria/2170/2025  A/Bulgaria/223/2025  A/Bulgaria/2269/2025  A/Bulgaria/228/2025  A/Bulgaria/244/2025  A/Bulgaria/2507/2025  A/Bulgaria/2540/2025  A/Bulgaria/2610/2025  A/Bulgaria/2650/2025  A/Bulgaria/279/2025  A/Bulgaria/293/2025  A/Bulgaria/3158/2024  A/Bulgaria/3179/2024  A/Bulgaria/3226/2024  A/Bulgaria/337/2025  A/Bulgaria/3372/2024  A/Bulgaria/3380/2024  A/Bulgaria/3479/2024  A/Bulgaria/3480/2024  A/Bulgaria/357/2025  A/Bulgaria/426/2025  A/Bulgaria/441/2025  A/Bulgaria/589/2025  A/Bulgaria/698/2025  A/Bulgaria/732/2025  A/Bulgaria/899/2025  A/Bulgaria/92/2025 | **Accession number**  EPI_ISL_17830834  EPI_ISL_20245744  EPI_ISL_20245766  EPI_ISL_20245769  EPI_ISL_20245773  EPI_ISL_20245775  EPI_ISL_19858891  EPI_ISL_19858889  EPI_ISL_19858878  EPI_ISL_19858876  EPI_ISL_19858887  EPI_ISL_20245843  EPI_ISL_19858874  EPI_ISL_20245746  EPI_ISL_20245747  EPI_ISL_19858885  EPI_ISL_19858872  EPI_ISL_20245782  EPI_ISL_20245783  EPI_ISL_20245748  EPI_ISL_20245749  EPI_ISL_19723142  EPI_ISL_19723149  EPI_ISL_19685487  EPI_ISL_20245751  EPI_ISL_19685482  EPI_ISL_19686982  EPI_ISL_19686980  EPI_ISL_19685602  EPI_ISL_20245752  EPI_ISL_20245753  EPI_ISL_20245754  EPI_ISL_20245756  EPI_ISL_20245757  EPI_ISL_20245758  EPI_ISL_20245841  EPI_ISL_20245745 | **A(H3N2) strains**  *A/Thailand/8/2022*  A/Bulgaria/1009/2025  A/Bulgaria/1122/2025  A/Bulgaria/1185/2025  A/Bulgaria/1285/2025  A/Bulgaria/1434/2025  A/Bulgaria/1474/2025  A/Bulgaria/1499/2025  A/Bulgaria/1511/2025  A/Bulgaria/1547/2025  A/Bulgaria/1588/2025  A/Bulgaria/1594/2025  A/Bulgaria/1632/2025  A/Bulgaria/1641/2025  A/Bulgaria/1691/2025  A/Bulgaria/1759/2025  A/Bulgaria/1868/2025  A/Bulgaria/1877/2025  A/Bulgaria/1894/2025  A/Bulgaria/1941/2025  A/Bulgaria/1944/2025  A/Bulgaria/2159/2025  A/Bulgaria/2334/2025  A/Bulgaria/2361/2025  A/Bulgaria/2397/2025  A/Bulgaria/2437/2025  A/Bulgaria/2503/2025  A/Bulgaria/2506/2025  A/Bulgaria/2509/2025  A/Bulgaria/2560/2025  A/Bulgaria/2768/2025  A/Bulgaria/2802/2025  A/Bulgaria/2825/2025  A/Bulgaria/298/2025  A/Bulgaria/3225/2024  A/Bulgaria/3356/2024  A/Bulgaria/3365/2024 | **Accession number**  EPI_ISL_19194107  EPI_ISL_20245742  EPI_ISL_20245743  EPI_ISL_20245764  EPI_ISL_20245768  EPI_ISL_20245770  EPI_ISL_20245771  EPI_ISL_20245772  EPI_ISL_20245268  EPI_ISL_20245774  EPI_ISL_20245776  EPI_ISL_20245777  EPI_ISL_20245778  EPI_ISL_20245780  EPI_ISL_20245781  EPI_ISL_19857417  EPI_ISL_19857415  EPI_ISL_19857412  EPI_ISL_19857414  EPI_ISL_19857410  EPI_ISL_19857413  EPI_ISL_19857411  EPI_ISL_19857409  EPI_ISL_19857408  EPI_ISL_19857406  EPI_ISL_19857407  EPI_ISL_19857404  EPI_ISL_19857402  EPI_ISL_19857403  EPI_ISL_19857405  EPI_ISL_19857400  EPI_ISL_19857401  EPI_ISL_20245800  EPI_ISL_20245750  EPI_ISL_19685597  EPI_ISL_19685598  EPI_ISL_19685600 |
| --- | --- | --- | --- |
| **B/Victoria lineage**  *B/Austria/1359417/2021*  B/Bulgaria/1111/2025  B/Bulgaria/1633/2025  B/Bulgaria/1719/2025  B/Bulgaria/1876/2025  B/Bulgaria/1918/2025  B/Bulgaria/2066/2025  B/Bulgaria/2125/2025  B/Bulgaria/2173/2025  B/Bulgaria/2270/2025  B/Bulgaria/2295/2025  B/Bulgaria/2394/2025  B/Bulgaria/2399/2025  B/Bulgaria/2463/2025  B/Bulgaria/2544/2025  B/Bulgaria/2620/2025  B/Bulgaria/2784/2025  B/Bulgaria/2848/2025  B/Bulgaria/2860/2025  B/Bulgaria/3150/2024  B/Bulgaria/3276/2024  B/Bulgaria/3538/2024  B/Bulgaria/3539/2024 | **Accession number**  EPI_ISL_19313795  EPI_ISL_20245763  EPI_ISL_20245779  EPI_ISL_19858985  EPI_ISL_19858984  EPI_ISL_19858983  EPI_ISL_19858982  EPI_ISL_19858981  EPI_ISL_19858980  EPI_ISL_19858979  EPI_ISL_19858978  EPI_ISL_19858977  EPI_ISL_19858976  EPI_ISL_19858975  EPI_ISL_19858974  EPI_ISL_19858973  EPI_ISL_19858972  EPI_ISL_20245761  EPI_ISL_20245762  EPI_ISL_19685614  EPI_ISL_19685616  EPI_ISL_19685618  EPI_ISL_19685619 | **A(H3N2) strains**  A/Bulgaria/3375/2024  A/Bulgaria/3483/2024  A/Bulgaria/3484/2024  A/Bulgaria/3485/2024  A/Bulgaria/3540/2024  A/Bulgaria/3541/2024  A/Bulgaria/3542/2024  A/Bulgaria/3568/2024  A/Bulgaria/3571/2024  A/Bulgaria/3618/2024  A/Bulgaria/3619/2024  A/Bulgaria/587/2025  A/Bulgaria/754/2025  A/Bulgaria/774/2025  A/Bulgaria/875/2025  A/Bulgaria/885/2025 | **Accession number**  EPI_ISL_19685601  EPI_ISL_19685605  EPI_ISL_19685607  EPI_ISL_19686973  EPI_ISL_19685608  EPI_ISL_19685609  EPI_ISL_19686970  EPI_ISL_19685610  EPI_ISL_19685611  EPI_ISL_19685613  EPI_ISL_19685488  EPI_ISL_20245755  EPI_ISL_20245759  EPI_ISL_20245760 EPI_ISL_20245740  EPI_ISL_20245741 |
