# Supplementary material for: Viral pathogens in the etiology of acute respiratory infections in Bulgaria during the 2024–2025 season and genetic diversity of circulating influenza viruses
Source: Front Microbiol. 2026 Apr 16;17:1785399. doi: 10.3389/fmicb.2026.1785399 (PMC13131023; doi:10.3389/fmicb.2026.1785399)
Supplement: Supplementary file 2 [file Table_2.docx]

**Supplementary Table 2** Amino acid substitutions identified in HA protein of influenza A(H1N1)pdm09, A(H3N2), and B/Victoria lineage viruses circulating in Bulgaria during the 2024-2025 season (without signal peptide)

| **Viruses/genetic clades/subclades** | **AA substitutions** | **Antigenic sites** | **Number of strains (%)** |
| --- | --- | --- | --- |
| ***A(H1N1)pdm09*** (n=36) | | | |
| All strains | R223Q |  | 36 (100) |
| C.1.9 | K43R |  | 2 (5.6) |
| D.5 | R45K |  | 1 (2.8) |
| C.1.9.4 | Q54K |  | 1 (2.8) |
| C.1.9.3 | S83P |  | 28 (77.8) |
| C.1.9.4 | D86N |  | 1 (2.8) |
| C.1.9; C.1.9.1, C.1.9.3, and C.1.9.4 | T120A |  | 35 (97.2) |
| C.1.9.4 | N125D | Sa | 1 (2.8) |
| C.1.9; C.1.9.3, and C.1.9.4 | S137P | Ca2 | 29 (80.6) |
| C.1.9; C.1.9.1, C.1.9.3, and C.1.9.4 | R142K | Ca2 | 35 (97.2) |
| C.1.9.4 | I149V |  | 1 (2.8) |
| C.1.9.3 | I166V | Ca1 | 15 (41.7) |
| C.1.9; C.1.9.1, C.1.9.3, and C.1.9.4 | K169Q | Ca1 | 35 (97.2) |
| C.1.9.3 | R205K | Ca1 | 8 (22.2) |
| C.1.9; C.1.9.1, C.1.9.3, and C.1.9.4 | A216T |  | 35 (97.2) |
| C.1.9; C.1.9.1, C.1.9.3, and C.1.9.4 | E260D |  | 35 (97.2) |
| C.1.9; C.1.9.1, C.1.9.3, and C.1.9.4 | A277T |  | 35 (97.2) |
| C.1.9; C.1.9.1, C.1.9.3, and C.1.9.4 | D356E |  | 35 (97.2) |
| C.1.9.3 | K402R |  | 2 (5.6) |
| C.1.9; C.1.9.1, C.1.9.3, and C.1.9.4 | H451N |  | 35 (97.2) |
| C.1.9 and C.1.9.3 | I510T |  | 29 (80.6) |
| C.1.9 | S529T |  | 2 (5.6) |
| ***A(H3N2)*** (n=49) | | | |
| All strains | Y195F |  | 49 (100) |
| J.2 | N8D | -CHO | 2 (4.1) |
| J.1.1 | R33Q |  | 2 (4.1) |
| J.2.2 | T65K | -CHO | 4 (8.2) |
| J.2 | N94K | E -CHO | 1 (2) |
| J.2 | S96I | -CHO | 1 (2) |
| J.2 | D104N |  | 5 (10.2) |
| J.2 and J.2.2 | N122D | A -CHO | 47 (95.9) |
| J.2.2 | S124N | A - CHO | 4 (7.1) |
| J.2 | T135A | A - CHO | 5 (10.2) |
| J.2 | T135K | A - CHO | 5 (10.2) |
| J.1.1, J.2, and J.2.2 | S145N | A | 13 (26.5) |
| J.2 and J.2.2 | I182V |  | 47 (95.9) |
| J.1.1 | I214T | D | 2 (4.1) |
| J.2 | V223I |  | 32 (65.3) |
| J.2 and J.2.2 | K276E |  | 47 (95.9) |
| J.2 | K278R | C | 27 (55.1) |
| J.1.1 | V347M |  | 2 (4.1) |
| ***B/Victoria lineage*** (n=22) | | | |
| All strains | D194E | 190-helix | 22 (100) |
| C.5.6.1 | T37I |  | 3 (13.6) |
| C.5.1 | E128K | 120-loop | 2 (9.1) |
| C.5.7 | E128G | 120-loop | 2 (9.1) |
| C.5.6.1 | E128D | 120-loop | 3 (13.6) |
| C.5.6 and C.5.6.1 | D129N | 120-loop | 18 (81.8) |
| C.5.1 and C.5.7 | E180K |  | 4 (18.2) |
| C.5.6 and C.5.6.1 | T196A | 190-helix | 18 (81.8) |
