# Supplementary material for: Viral pathogens in the etiology of acute respiratory infections in Bulgaria during the 2024–2025 season and genetic diversity of circulating influenza viruses
Source: Front Microbiol. 2026 Apr 16;17:1785399. doi: 10.3389/fmicb.2026.1785399 (PMC13131023; doi:10.3389/fmicb.2026.1785399)
Supplement: Supplementary file 3 [file Table_3.docx]

**Supplementary Table 3** Amino acid substitutions identified in NA protein of influenza viruses A(H1N1)pdm09, A(H3N2) and B/Victoria lineage circulating in Bulgaria during the 2024-2025 season

| **Viruses/genetic clades/subclades** | **AA substitution** | **Number of strains (%)** |
| --- | --- | --- |
| ***A(H1N1)pdm09* (n=36)** | | |
| All strains | D50N | 36 (100) |
|  | S200N | 36 (100) |
|  | E382G | 36 (100) |
| C.1.9.1 | T19M | 2 (5.6) |
| C.1.9.3 | I46V | 22 (61.1) |
| C.1.9 and C.1.9.3 | S52N - CHO | 29 (80.6) |
| C.1.9.3 | A86E | 3 (8.3) |
| C.1.9.3 | A86T | 2 (5.6) |
| C.1.9.3 | S89A | 3 (8.3) |
| C.1.9.3 | S153N | 1 (2,8) |
| C.1.9.3 | V234I | 8 (22.2) |
| C.1.9.3 and D.5 | I263V | 4 (11.1) |
| C.1.9; C.1.9.1, C.1.9.3, and C.1.9.4 | I264T | 34 (94.4) |
| ***A(H3N2) (*n=47)** | | |
| All strains | K210R | 47 (100) |
| J.2 | P45S | 3 (6.4) |
| J.2.2 | A82V | 4 (8.5) |
| J.1.1 | E83G | 2 (4.3) |
| J.2 | G93S | 4 (8.5) |
| J.2 | L140I | 5 (10.6) |
| J.2 | I307M | 25 (53.2) |
| J.1.1 | R340K | 2 (4.3) |
| J.2 | N393K | 3 (6.4) |
| J.1.1, J.2 and J.2.2 | R400K | 20 (42.6) |
| J.2.2 | S416N | 4 (8.5) |
| ***B/Victoria lineage* (n=22)** | | |
| All strains | I459V | 22 (100) |
| C.5.6 and C.5.6.1 | I45T | 18 (81.8) |
| C.5.7 | A47V | 2 (9.1) |
| C.5.1 | L52S | 2 (9.1) |
| C.5.6 | R65L | 14 (63.6) |
| C.5.6 | V193I | 13 (59,1) |
| C.5.6 | K360N | 2 (9.1) |
| C.5.1 and C.5.7 | G378E | 4 (18.2) |
| C.5.1 | G384D | 2 (9.1) |
| C.5.6 and C.5.6.1 | S397N | 18 (81.8) |
| C.5.6 and C.5.6.1 | K404R | 18 (81.8) |
