# Supplementary material for: Viral pathogens in the etiology of acute respiratory infections in Bulgaria during the 2024–2025 season and genetic diversity of circulating influenza viruses
Source: Front Microbiol. 2026 Apr 16;17:1785399. doi: 10.3389/fmicb.2026.1785399 (PMC13131023; doi:10.3389/fmicb.2026.1785399)
Supplement: Supplementary file 4 [file Table_4.docx]

**Supplementary Table 4** Amino acid substitutions identified in PB2, PB1, PA, NP, MP, and NS proteins of influenza viruses A(H1N1)pdm09, A(H3N2), and B/Victoria lineage circulating in Bulgaria during the 2024-2025 season

| Segments | Viral genes/ proteins | Influenza A(H1N1)pdm09 | Influenza A(H3N2) | Influenza B/Victoria |
| --- | --- | --- | --- | --- |
| 1 | PB2 | D9N, I81M, K113T, K299R, L475M | N79S, D107N, I147T, V410M | I396M |
| 2 | PB1 | K57T, K757R | 0 | D51N, M65I, A387T,  I462V |
| 3 | PA | V14I, M61I, L63I, R262K, K339N,K716E | G101E, N142K, I311M, T402S, K605R | I376M, S530T |
| 5 | NP | E14D, T22A, I33V, I136L, V217I, Q453P | M136L, D220E, R236K, I418L | A28T, I445M |
| 7 | M1 | 0 | K210R | R105K, N221S |
| 8 | NS1 | S212P | K26N, L33I, V60A, V82A, M124I, I171V, H207N | Y104H, A212V, V263M |
